# Post-natal gestational age assessment using targeted metabolites of neonatal heel prick and umbilical cord blood: A GARBH-Ini cohort study from North India

**DOI:** 10.7189/jogh.14.04115

**Published:** 2024-07-05

**Authors:** Thirunavukkarasu Ramasamy, Bijo Varughese, Mukesh Singh, Pragya Tailor, Archana Rao, Sumit Misra, Nikhil Sharma, Koundiya Desiraju, Ramachandran Thiruvengadam, Nitya Wadhwa, GARBH-Ini Study Group, Seema Kapoor, Shinjini Bhatnagar, Pallavi Kshetrapal

**Affiliations:** 1 Lab of Perinatal Research, Maternal and Child Health, Translational Health Science and Technology Institute, Faridabad, Haryana, India; 2 Genetics Laboratory, Department of Paediatrics, Maulana Azad Medical College, New Delhi, India; 3 Department of Gastroenterology, All India Institute of Medical Sciences, New Delhi, India; 4 Gurugram Civil Hospital, GCH, Haryana, India; 5 Maternal and Child Health, Translational Health Science and Technology Institute, Faridabad, Haryana, India; 6 Interdisciplinary Group for Advanced Research on Birth Outcomes - DBT India Initiative, Translational Health Science and Technology Institute, Faridabad, Haryana, India

## Abstract

**Background:**

Accurate assessment of gestational age (GA) and identification of preterm birth (PTB) at delivery is essential to guide appropriate post-natal clinical care. Undoubtedly, dating ultrasound sonography (USG) is the gold standard to ascertain GA, but is not accessible to the majority of pregnant women in low- and middle-income countries (LMICs), particularly in rural areas and small secondary care hospitals. Conventional methods of post-natal GA assessment are not reliable at delivery and are further compounded by a lack of trained personnel to conduct them. We aimed to develop a population-specific GA model using integrated clinical and biochemical variables measured at delivery.

**Methods:**

We acquired metabolic profiles on paired neonatal heel prick (nHP) and umbilical cord blood (uCB) dried blood spot (DBS) samples (n = 1278). The master data set consists of 31 predictors from nHP and 24 from uCB after feature selection. These selected predictors including biochemical analytes, birth weight, and placental weight were considered for the development of population-specific GA estimation and birth outcome classification models using eXtreme Gradient Boosting (XGBoost) algorithm.

**Results:**

The nHP and uCB full model revealed root mean square error (RMSE) of 1.14 (95% confidence interval (CI) = 0.82–1.18) and of 1.26 (95% CI = 0.88–1.32) to estimate the GA as compared to actual GA, respectively. In addition, these models correctly estimated 87.9 to 92.5% of the infants within ±2 weeks of the actual GA. The classification models also performed as the best fit to discriminate the PTB from term birth (TB) infants with an area under curve (AUC) of 0.89 (95% CI = 0.84–0.94) for nHP and an AUC of 0.89 (95% CI = 0.85–0.95) for uCB.

**Conclusion:**

The biochemical analytes along with clinical variables in the nHP and uCB data sets provide higher accuracy in predicting GA. These models also performed as the best fit to identify PTB infants at delivery.

Preterm birth (PTB) is defined as a live-born baby delivered before 37 completed weeks of gestation [1,2]. It is one of the major global pregnancy complications with more than 60% of PTB occurring in lower-middle-income countries (LMICs) [3,4]. Preterm birth, small for gestational age (SGA) and low birth weight (LBW) newborns have been categorised as small vulnerable newborns (SVNs) under a new conceptual framework. The rate of SVNs has been reported to be the highest in South Asia and sub-Saharan Africa [5,6]. Global Network’s population-based registry of pregnant women in LMICs has reported the overall PTB rate to be 12.6% [7]. In India, ~ 3.5 million babies are born PTB every year that contributes to the largest percentage (23.5%) of the global burden of 15 million born as PTB annually. Preterm birth is one of the leading causes of death among children under five years of age with adverse consequences beyond early infancy and later in life resulting in major human and economic loss [8,9]. A recent systematic analysis presents India with the highest number of PTB in 2020 (3.02 million) that accounts for over 20% of all PTB worldwide [1]. The PTB rates of 13.5%, based on dating ultrasonography (USG) have been documented systematically for the first time in India from a longitudinal large pregnancy cohort in North India [10].

In most LMICs, estimating GA at or after delivery is highly challenging; more so, in un-booked pregnancies [11]. Accurate GA is essential for timely decisions on effective therapeutic interventions at birth and documenting the developmental milestones of neonates. Currently, assessing the true GA postnatally in a clinical setting is based on birthweight, which may be affected by ethnicity, socioeconomic status, living conditions, and natural environment [12,13]. Gestational age is also determined at delivery using the last menstrual period which has also been reported to be unreliable due to recall bias or irregular periods [14]. Other postnatal GA assessment methods currently practiced by neonatologists such as Dubowitz and New Ballard score system have poor reliability and high inter-user variability [15–17]. Examination by USG is the only proven reliable method to assess the GA accurately but has limited accessibility in most of the LMICs settings. High equipment costs and lack of trained manpower further prohibit its use in most clinical settings. Thus, there is a need for a novel, feasible, and cost-effective diagnostic tool for post-natal GA assessment.

Pregnancy is a phase of steady physiologic adaptation that affects nutrient metabolism [18]. Multiple metabolites accessed in maternal plasma longitudinally across pregnancy revealed that metabolic clock of five blood metabolites accurately predict GA [19]. Reports from mid-trimester plasma have associated maternal metabolites with PTB delivery [20]. In addition, to modulation of amino acid and fatty acid metabolites in maternal circulation during pregnancy, alterations in umbilical cord blood (uCB) metabolites collected at delivery have been found to be associated with PTB and LBW. Besides maternal and uCB metabolites, several prediction models using metabolic markers from neonatal heel prick (nHP) collected during routine newborn screening (NBS) have been developed to determine GA at birth [21,22]. External validation of these models in other global populations has demonstrated satisfactory performance in accurately predicting GA and SGA [23–25]. However, these models also over or under-estimated GA in PTB and TB infants [24] respectively. To the best of our knowledge, no studies have reported development of a post-natal GA prediction model based on paired nHP and uCB samples from the same participant and its use in discriminating PTB in an Indian population. We included the uCB in the development of the metabolic models due to the enhanced acceptability of metabolites testing and ease in sample collection methods both for the family members and health care providers.

This study aims to develop a population-specific machine learning (ML) prediction model within an ongoing pregnancy cohort coordinated by the interdisciplinary Group for Advanced Research on BirtH outcomes-DBT India Initiative (GARBH-Ini). Metabolites from paired uCB and nHP samples were measured using mass-spectometry platforms at a newborn clinical facility. GA estimation and birth outcome classification models have been developed using XGBoost algorithm. These models would be crucial to accurately assess GA at delivery for immediate clinical care of adverse birth outcomes. Besides these models may aid as surveillance tools to inform region-specific PTB rates in LMICs.

## METHODS

### Study cohort and participants

**Figure 1:**
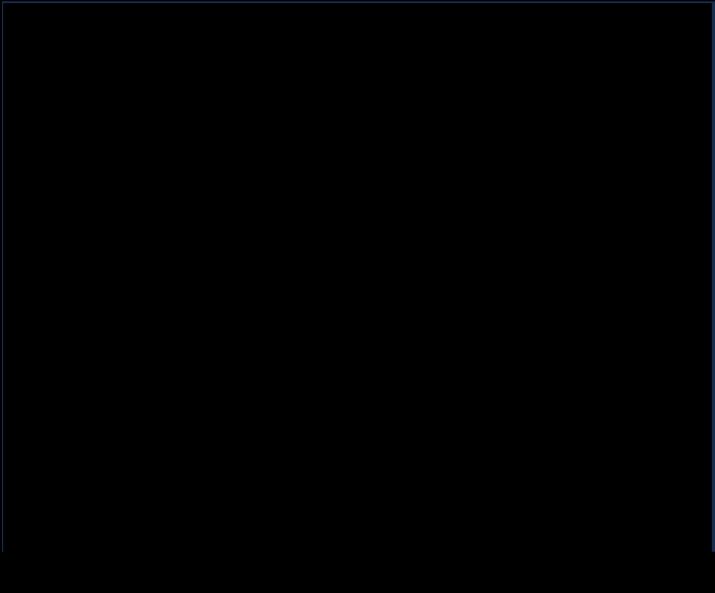
Screening and selection of study population for nHP and uCB samples from the GARBH-Ini cohort. *Samples collected between 31 May 2017 and 31 December 2019. nHP – neonatal heel prick, uCB – umbilical cord blood

The GARBH-Ini cohort is an ongoing prospective observational hospital-based pregnancy cohort at the district hospital in Gurugram, North India. Detailed information can be assessed from our previous study [10]. Briefly, pregnant women are enrolled after providing informed consent at <20 weeks of gestation determined by dating USG as per the World Health Organization criteria [26]. Those women are followed at regular intervals to document their clinical, medical history, socio-demographic characteristics, and pregnancy outcomes. Maternal and neonatal samples were collected and processed at the clinical site and bio-banked at the institutional biorepository. Institutional ethics approvals have been obtained from all participating organisations. Participants for this study were selected from the mothers who delivered singleton live births and had provided paired nHP and uCB samples. Detailed information on the selection of samples used to develop the population-specific model for the GARBH-Ini cohort is presented in **Figure 1**. Figure S1 in the **Online Supplementary Document** presents the overview of the study design.

### Sample collection and processing

The uCB was collected immediately after delivery and nHP sample, after 20 hours but within 72 hours post-delivery was spotted on Protein Saver 903 cards. These cards were air-dried and stored at the institutional biorepository at –80°C as described previously [10]. The qualified dried blood spot (DBS) Protein saver 903 cards (nHP and uCB) were shipped on dry ice to the Pediatrics Research and Genetic NBS laboratory established as a part of Government initiative for Neonatal Early Evaluation Vision, at Maulana Azad Medical College, New Delhi. Using DBS Panthera auto-puncher (PerkinElmer, Inc.), the discs were punched from each sample for testing of the following analytes: thyroid-stimulating hormone (TSH); 17-O hydroxyprogesterone (17-OHP), galactose-1-phosphate uridylyltransferase (GALT), 11 amino acids and 30 acylcarnitines. These samples were subjected to extraction of the above biochemical analytes using NeoBase Non-derivatized MSMS kit (PerkinElmer, USA) according to manufacturer instructions. The neonatal 17-OHP and TSH were measured using PerkinElmer AutoDELFIA Immunoassays (PerkinElmer); GALT level was measured using the GSP® Instrument (PerkinElmer), and amino acids and acylcarnitine analysis were performed using flow injection analysis- tandem mass spectrometry (FIA-tandem-MS) (SCIEX, USA) (Table S1 in the **Online Supplementary Document**).

### Data pre-processing and statistical analysis

Clinical and biochemical analytes were compiled as a master data set. The missing value of the biochemical parameters was imputed using the k-nearest neighbor (KNN) algorithm [27]. Birth outcomes such as SGA (<10th percentile), appropriate for GA (AGA, 10th to 90th percentile), and large for GA (LGA, >90th percentile) were determined based on the INTERGROWTH-21st standardised growth charts [28]. To minimise the outlier influence and reduce skewness, the data set was scaled by standard score. Mean and standard deviation (SD) was described for continuous variables. Categorical variables were described as percentages and proportions. Univariate analysis was performed to compare the baseline characteristics between PTB and TB infant groups using *t*-tests and Chi-squared tests for continuous and categorical variables, respectively.

### Feature selection and its implementation for model development

The nHP and uCB data sets consisted of 47 predictor variables including 44 measured biochemical analytes and three clinical factors (birthweight, placental weight, and gender of the baby (Table S1 in the **Online Supplementary Document**). For implementing the development of machine learning (ML) algorithms, the data set was randomly split into training (70%) and testing (30%) subsets for both the nHP and uCB data sets. To remove multicollinearity and to improve the model accuracy, we performed feature selection on predictor variables against GA as a continuous dependent variable on 70% of the training data set to obtain the best predictors using the Boruta package [29]. The confirmed and tentative variables were selected and the unconfirmed variables with the hit score <0.5 were omitted from further analysis. A *t*-test was performed to compare the mean differences between PTB and TB infants in nHP and uCB data sets. With the help of selected predictors, six different models were developed for estimating the post-natal GA and also for discriminant analysis of the PTB from TB as described (Table S3 in the **Online Supplementary Documents**). To address the nonlinearity between dating USG GA and each confirmed variable, all the linear predictors were squared. The linear and squared terms of selected predictors were used in the model-building data set (Table S1 in the **Online Supplementary Document**).

### Selection of ML algorithm

For implementing the ML algorithms, the selected predictors were used for the data sets. A 10-fold cross-validation method with three repeats was used as a resampling procedure on the training data set to evaluate and select the ML algorithms [30]. Five ML algorithms: Linear regression (LR), random forest (RF), Elastic net (EN), linear support vector machines (L-SVM), and XGBoost were used to achieve the best model [31,32].

### Implementation of XGBoost algorithm for model development

XGBoost algorithm, a decision tree-based ensemble algorithm has been widely used for diagnosis and prognosis as a predictive tool in the medical field [33–35]. For better prediction of GA using biochemical analytes along with placental weight, and birth weight, the XGBoost was employed. It is a robust algorithm capable of a high degree of accuracy and interpretability [36]. The regularisation parameters used for calibration of the model prevent overfitting of the data sets. Moreover, every tree learns from the residuals of all previous trees in XGBoost. To attempt to improve predictive performance and find a balance between bias and variance, the following hyper-parameters were optimised such as 1) resampling method (repeatedcv = 10 with 3 repeats as constant) and 2) tuneGrid parameters (n_rounds, eta, max_depth, gamma, colsample_bytree, subsample, and min_child_weight). The XGBoost regression (reg: squared error) was tuned for estimating the GA as a continuous outcome and as a classifier function (binary: logistic) to discriminate the PTB from TB. All the above algorithms and parameters were implemented using XGBoost with essential dependency packages in the R programme (R Core Team, Statistical Computing, Vienna, Austria).

### Performance of the population-specific GA models

Initially, the final algorithm was tested for all the models to predict the GA using the test data set (n = 383). The performance of the models was assessed by estimating root mean squared error (RMSE) and mean absolute error (MAE) against actual GA (dating USG). RMSE and MAE as units of GA, were used to access the optimal model in the test data set. In addition, the proportion of estimated GA of infants correctly within 1, 2, 3, and 4 weeks of actual GA was also assessed. The XGBoost has also been used in several studies to predict the probability of a binary outcome (such as ‘yes’ or ‘no’). The binary outcome was converted to a probability of 0-1 for PTB (<37 weeks of GA) and TB (≥37 weeks of GA) with the help of the pROC package [37]. A threshold value was then chosen to classify the observations into two classes. If the predicted probability is greater than the threshold value (*P* > 0.5), the observation is classified as PTB, otherwise, it is classified as TB. The area under the receiver operator curve (AUC) was calculated through sensitivity and specificity for prediction of PTB<37 vs. TB≥37 weeks against the gold standard GA measured by dating USG [38]. To understand the GA model performances based on the birth outcomes, the test data was stratified into five different sub-groups, such as SGA, non-SGA, birth weight (<2500 g), PTB, and TB.

## RESULTS

### Baseline characteristics of the study population

A total of 2556 DBS samples comprising 1278 paired nHP and uCB respectively were selected for the current study. The baseline characteristics of the study population are presented in **Table 1**. The distribution of TB and PTB included in the study was 1181 and 97, respectively. The ratio of the male and female neonates was almost 1:1. Gestational age as confirmed by dating USG was 39.03 ± 1.8 weeks for TB and 35.6 ± 1.1 for PTB. Among neonates who weighed <2500 g, the frequency of PTB was higher (73.2%) as compared to TB (19.1%). Placental weight and birth weight were higher in the babies born term than those born preterm. Using the intergrowth charts, our study population reflected 37.8% SGA, 38.9% term SGA, and 60.2% term non-SGA. Among the PTB group, 24.7% were SGA and the remaining were non-SGA.

**Table 1:** Baseline characteristics of the study population selected for postnatal-GA model development

Characteristics	Overall (n = 1278)	Term birth (n = 1181)	Preterm birth (n = 97)
Maternal age (mean ± SD)	23.7 ± 3.8	23.7 ± 3.7	23.7 ± 3.9
Sex, n (%)			
*Male*	662 (51.7)	606 (51.3)	56 (57.7)
*Female*	616 (48.3)	575 (48.7)	41 (42.3)
GA (mean ± SD)	39.03 ± 1.50	39.3 ± 1.8	35.62 ± 1.1
Birth weight (g), (mean ± SD)	2793.26 ± 424.0	2839.9 ± 398.2	2266.3 ± 350.3
Birth weight group, n (%)			
*≥2500 g*	981 (76.8)	955 (80.9)	26 (26.8)
*<2500 g*	297 (23.2)	226 (19.1)	71 (73.2)
Placental weight, mean ± SD	488.4 ± 109.7	491.4 ± 109.4	454.3 ± 107.7
Size for GA (n, %)			
*SGA*	483 (37.8)	459 (38.9)	24 (24.7)
*AGA*	784 (61.3)	711 (60.2)	73 (75.3)
*LGA*	11 (0.8)	11 (0.9)	0 (0)

AGA – average for gestational age, LGA – large for gestational age, GA – gestational age, SD – standard deviation, SGA – small for gestational age

### Selection of predictors for model building

Biochemical analytes including the amino acids (n = 11), acylcarnitines (n = 30), enzymes (n = 1), and hormones (n = 2) were measured as per standard NBS procedures on 1278 paired nHP and uCB DBS cards. Along with these analytes, clinical data on baby gender, birth weight, and placental weight were also included in the model development data set (Table S1 in the **Online Supplementary Document**). Using the curated data set of these 47 features, a wrapper-based approach for feature selection (Boruta algorithm) resulted in 31 and 24 predictors for the development of the post-natal GA model from nHP and uCB data sets, respectively (Figures S2A–B in the **Online Supplementary Document**). Among 31 selected predictors in the nHP data set, eight analytes (two amino acids and six acylcarnitines), and two clinical factors (placental weight and birth weight) were significantly different between PTB and TB infants. Of the 24 selected predictors in the uCB data set, nine analytes (one hormone, two amino acids, and six acylcarnitines) and two clinical factors (birth weight and placental weight) were significantly different between these two groups (Tables 2A–B in the **Online Supplementary Document**). Interestingly, five analytes (alanine, C2, C4DC, C5, and C14:1) were common both in the nHP and uCB data set that identified significant differences between PTB and TB infants.

### Postnatal GA models and their performance in the overall test data set

Using the selected predictors, six models were built on the training data set (Table S3 in the **Online Supplementary Document**). Although these models were trained using five robust ML algorithms, the XGBoost was taken forward as the best fit robust algorithm for testing these population-specific models. When the XGBoost algorithm was applied to the data sets, birthweight only model (M1) revealed an RMSE of 1.3 (95% confidence interval (CI) = 0.91–1.38) and MAE of 1.01 (95% CI = 0.91–1.28). As compared to this, baseline clinical model, that consists of placental weight and birth weight, (M2) revealed a slightly better GA estimation performance, with RMSE of 1.28 (95% CI = 0.89–1.35) and MAE of 1.01 (95% CI = 0.9–1.26). Among the metabolites only models nHP analyte-only model (M3) provided better estimation performance (RMSE = 1.33 with 95% CI = 0.96–1.37; MAE = 1.07 with 95% CI = 0.96–1.35) than uCB analyte only model (M4) (RMSE = 1.46 with 95% CI = 1.03–1.48; MAE = 1.13 with 95% CI = 0.99–1.41). When the clinical factors were combined with the analytes, in the nHP full model (M5), a more accurate GA (RMSE = 1.14 with 95% CI = 0.82–1.18; MAE = 0.9 with 95% CI = 0.81–1.11) as compared to the uCB full model (M6) (RMSE = 1.26 with 95% CI = 0.88–1.32; MAE = 0.98 with 95% CI = 0.86–1.13) was attained. This was the best when compared to any of the other above-indicated models. However, the uCB full model (M6) did perform better than the baseline clinical (M2) and analyte-only models (M4).

**Figure 2:**
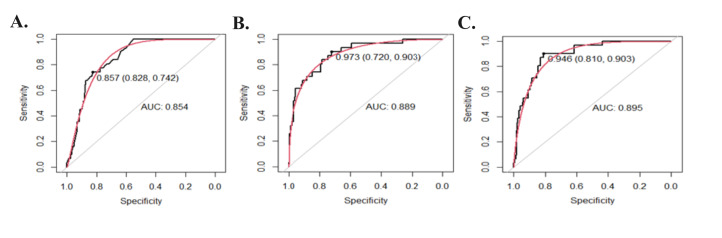
Performance of models in correctly classifying the infants based on PTB discrimination threshold (<37 weeks of GA) on test data set. ROC curve analysis for birth weight model (**Panel A**), nHP full model (**Panel B**), and uCB full model with their respective AUC (**Panel C**). AUC – area under the curve, nHP – neonatal heel prick, PTB – preterm birth, ROC – receiver operating characteristic, uCB – umbilical cord blood

The ability to correctly classify PTB from TB by receiver operating characteristic (ROC) analysis by calculating the AUC in the six models is shown in the **Table 2** and Table S4 in the **Online Supplementary Document**. Of the six models analysed, three models performed very well (**Figure 2**, panels A–C). The birthweight-only model (M1) reported an AUC of 85% (95% C I = 0.80–0.90) and the nHP full model (M5) showed an AUC of 89% (95% CI = 0.84–0.94) that was similar to the uCB full model (M6) (AUC = 89%; 95% CI = 0.84–0.94). The baseline clinical model (M2) (AUC = 87%; 95% CI = 0.81–0.92) performed better than the birthweight-only model (M1). The analyte-only models (M3 and M4) did not perform well when compared to the other four models.

**Table 2:** Overall model performance for GA estimation and birth outcome as preterm birth and term birth discrimination on test data sets

Model	No. of predictors (linear, squared)	RMSE (95% CI)*	MAE (95% CI)*	AUC (95% CI)
Baby weight (M1)	2 (1, 1)	1.30 (0.91–1.38)	1.01 (0.91–1.28)	0.85 (0.81–0.91)
Baseline clinical (M2)	4 (2, 2)	1.28 (0.89–1.38)	1.01 (0.90–1.26)	0.87 (0.82–0.92)
Neonatal heel prick (M3)	58 (29, 29)	1.33 (0.96–1.37)	1.07 (0.96–1.35)	0.73 (0.64–0.82)
Umbilical cord blood (M4)	44 (22, 22)	1.46 (1.03–1.48)	1.13 (0.99–1.41)	0.69 (0.59–0.78)
Heel prick full model (M5)	62 (31, 31)	1.14 (0.82–1.18)	0.91 (0.81–1.11)	0.89 (0.84–0.94)
Umbilical Cord blood full model (M6)	48 (24, 24)	1.26 (0.88–1.32)	0.98 (0.86–1.13)	0.89 (0.85–0.95)

AUC – area under roc curve, CI – confidence interval, GA – gestational age, MAE – mean absolute error, RMSE – root mean squared error

*Data is presented as the mean and 5th and 95th bootstrap percentiles. GA for 1000 bootstrap samples generated from each data set.

### Frequency of difference in weeks between predicted and actual GA on the overall test data sets

The frequency of difference in weeks between predicted and actual GA is presented in **Table 3**. The nHP full model (M5) accurately separated 245 (65.5%), and 346 (92.5%) of infants within ±1 week and ±2 weeks, respectively. The baseline clinical model (M2) classified 225 (60.2%) and 332 (88.8%) infants within ±1 week and ±2 weeks respectively which was less predictive than nHP full model (M5) and slightly better than the uCB full model (M6); 329 (87.9%) within ±2 weeks. The baseline clinical (M2) and nHP analyte-only model (M3) revealed 332 (88.8%) of infant separation as an equal contribution for both the models within ±2 weeks. The nHP full model (M5) performed the best to classify the maximum number of infants within ±2 weeks (**Table 3**). The cross-tabulation between the actual and predicted GA in 2 weeks category interval in nHP and uCB full models, indicated the accuracy of the performance of these models (Table S5 in the **Online Supplementary Document**).

**Table 3:** Summary of model performance as the difference in weeks between predicted and dating ultrasound GA, as gold standard, on test data sets

Model	Difference*	0 week	1 week	2 weeks	3 weeks	≥4 weeks
M1	n (%)	12 (3.2)	218 (58.3)	99 (26.5)	36 (9.6)	9 (2.4)
	Cum (%)	12 (3.2)	230 (61.5)	329 (88.0)	365 (97.6)	374 (100)
M2	n (%)	10 (2.7)	215 (57.5)	107 (28.6)	32 (8.6)	10 (2.7)
	Cum (%)	44 (4.9)	225 (60.2)	332 (88.8)	364 (97.3)	374 (100)
M3	n (%)	8 (2.1)	208 (55.6)	116 (31.1)	35 (9.3)	7 (1.9)
	Cum (%)	8 (2.1)	216 (57.7)	332 (88.8)	367 (98.1)	374 (100)
M4	n (%)	8 (2.1)	204 (54.6)	109 (27.8)	44 (11.8)	14 (3.7)
	Cum (%)	8 (2.1)	212 (56.7)	316 (84.5)	360 (96.3)	374 (100)
M5	n (%)	9 (2.4)	236 (63.1)	101 (27.0)	25 (6.7)	3 (0.8)
	Cum (%)	9 (2.4)	245 (65.5)	346 (92.5)	371 (99.2)	374 (100)
M6	n (%)	15 (4.0)	213 (56.9)	101 (27.0)	37 (9.9)	8 (2.2)
	Cum (%)	15 (4.0)	228 (60.9)	329 (87.9)	366 (97.8)	374 (100)

GA – gestational age

*n (%) = No. of observation (%); Cum (%) = Cumulative (%).

### Performance of GA estimation model on test data sets stratified by birth outcomes

In the test data sets stratified as per birth outcomes, the term birth, non-SGA for all the models accurately estimated the GA better than PTB, birth weight <2500 g, and SGA subgroups (**Table 4**). Between all the five subgroups, the Model-5 had the least prediction error in TB (RMSE of 1.05 with 95% CI = 0.97–1.13; MAE of 0.78 with 95% CI = 0.41–0.76) and non-SGA (RMSE of 1.09 with 95% CI = 0.99–1.22; MAE of 0.87 with 95% CI = 0.62–0.89). The SGA group with an RMSE of 1.22 with 95% CI = 1.09–1.33; MAE of 1.01 with 95% CI = 0.88–1.15, birth weight <2500 g group with an RMSE of 1.42 with 95% CI = 1.23–1.59; MAE of 1.17 with 95% CI = 0.83–1.21 and PTB group with RMSE of 1.88 with 95% CI = 1.56–2.19; MAE of 1.21 with 95% CI = 0.95–1.24, are presented in **Table 4**. The Model-5 estimated 69.3% within ±1 week in non-SGA and 93.9% within ±2 weeks in TB as the highest percentage ranging between ±1 and ±2 week to the reference GA. For the rest of the subgroup models, details of GA estimated percentage within ±1 and ±2 weeks of reference GA are presented in **Table 4**.

**Table 4:** Summary of model performance to estimate GA from nHP and uCB samples on five different test data sets stratified by birth outcomes

	Model-1	Model-2	Model-3	Model-4	Model-5	Model-6
**SGA (n = 136)**						
RMSE (95% CI)*	1.38 (1.24–1.55)	1.42 (1.26–1.61)	1.28 (1.15–1.64)	1.4 (1.23–1.58)	1.22 (1.09–1.33)	1.42 (1.25–1.58)
MAE (95% CI)*	1.12 (0.85–1.19)	1.14 (0.86–1.21)	1.07 (0.83–1.11)	1.12 (0.79–1.07)	1.1 (0.88–1.15)	1.12 (0.81–1.09)
n (%) within ±1 week	67 (49.3)	69 (50.7)	71 (52.2)	71 (52.2)	73 (53.7)	76 (55.9)
n (%) within ±2 weeks	115 (84.6)	114 (83.8)	119 (87.5)	117 (86.1)	122 (89.7)	110 (80.9)
**Birth weight <2500 g (n = 91)**						
RMSE (95% CI)*	1.7 (1.43–1.96)	1.65 (1.44–1.86)	1.73 (1.45–1.99)	1.93 (1.61–2.24)	1.42 (1.23–1.59)	1.57 (1.36–1.78)
MAE (95% CI)*	1.28 (0.89–1.31)	1.29 (0.91–1.32)	1.48 (1.06–1.49)	1.68 (1.31–1.65)	1.17 (0.83–1.21)	1.30 (1.09–1.32)
n (%) within ±1 week	38 (41.8)	40 (43.9)	40 (43.9)	39 (42.9)	43 (47.3)	43 (47.3)
n (%) within ±2 weeks	71 (78.1)	71 (78.1)	70 (76.9)	65 (71.4)	76 (83.5)	69 (75.8)
**Preterm birth (n = 31)**						
RMSE (95% CI)*	2.3 (1.95–2.76)	2.18 (1.89–2.48)	2.72 (2.34–3.13)	3.05 (2.87–3.4)	1.88 (1.56–2.19)	2.19 (1.65–2.31)
MAE (95% CI)*	1.91 (1.44–1.99)	1.87 (1.41–1.93)	1.98 (1.62–2.07)	2.71 (2.41–2.82)	1.21 (0.95–1.24)	1.71 (1.41–1.85)
n (%) within ±1 week	3 (9.7)	3 (9.7)	2 (6.4)	0 (0)	6 (19.3)	4 (12.9)
n (%) within ±2 weeks	15 (48.4)	17 (54.8)	11 (35.5)	6 19.3)	22 (71.1)	20 (64.5)
**Non-SGA (n = 238)**						
RMSE (95% CI)*	1.25 (1.11–1.39)	1.19 (1.08–1.31)	1.37 (1.22–1.53)	1.51 (1.33–1.65)	1.09 (0.99–1.22)	1.18 (1.07–1.29)
MAE (95% CI)*	0.96 (0.82–1.09)	0.86 (0.76–0.98)	0.98 (0.73–0.94)	1.27 (1.09–1.34)	0.87 (0.62–0.91)	0.88 (0.77–0.99)
n (%) within ±1 week	149 (62.6)	153 (64.3)	133 (55.9)	127 (53.4)	165 (69.3)	144 (60.5)
n (%) within ±2 weeks	211 (88.6)	217 (91.2)	208 (87.4)	200 (84.1)	222 (93.3)	217 (91.2)
**Term birth (n = 343)**						
RMSE (95% CI)*	1.16 (1.07–1.25)	1.16 (1.07–1.27)	1.14 (1.06–1.21)	1.23 (1.15–1.33)	1.05 (0.97–1.13)	1.19 (1.09–1.28)
MAE (95% CI)*	0.79 (0.68–0.91)	0.75 (0.61–0.88)	0.82 (0.68–0.83)	0.96 (0.84–0.98)	0.78 (0.41–0.67)	0.96 (0.83–1.2)
n (%) within ±1 week	213 (62.1)	219 (63.8)	202 (58.9)	198 (57.7)	232 (67.6)	216 (63.1)
n (%) within ±2 weeks	311 (90.7)	314 (91.5)	316 (92.1)	311 (90.7)	322 (93.9)	307 (89.5)

CI – confidence interval, MAE – mean absolute error, RMSE – root mean square error, SGA – small for gestational age

*Data is presented as the mean and 5th and 95th bootstrap percentiles for MAE, RMSE and the percentage of model estimates within 1 and 2 weeks of ultrasound. GA for 1000 bootstrap samples generated from each data set.

## DISCUSSION

In this study, we have established a population-specific post-natal GA estimation model using targeted metabolomics. Several ML-based models have been implemented in the field of maternal and child health towards predicting pregnancy outcomes [33–35]. Lately, the XGBoost has emerged as one of the most powerful ensemble based ML algorithm and has been used for the better prediction of pregnancy complications and birth outcomes such as pre-eclampsia, gestational diabetes mellitus and growth restriction [39–41]. Out of the five algorithms we evaluated, the tree-based XGBoost algorithm had the best fit for accurately estimating post-natal GA. It is one of the most preferred and widely accepted ML algorithms for regression and classification tasks. Due to its high accuracy, scalability, regularisation, feature importance, and fast computational skills, has been utilised for building predictive models [36]. This algorithm not only helps in estimating the GA accurately but is also efficient in differentiating PTB from TB infants at delivery.

Before building our population-specific model, we validated the western-based GA model reported by the IOWA study group [21] on our data sets. We found trends of overestimation of the GA in weeks when compared to the actual GA tested against the overall study population. A study also reported overestimation of GA among the PTB group in the Asian and African populations using the IOWA model [24]. This could be due to differences in diet and ethnicity which are known to influence circulatory metabolites in newborns that have been reported to be highly associated with GA [21,42].

We present the development of six different population-specific models established to achieve the best model with high accuracy in estimating the post-natal GA. In our study, the birth weight-only model is able to estimate GA within 1.3 weeks, as an error rate compared to actual GA. Moreover, this model marginally enhances its accuracy while adding placental weight (baseline clinical model) as one of the predictors. These two models are equally comparable to the previously reported models [21,22,24,43]. Our baseline clinical model performs similarly to the earlier reported IOWA model developed on 51 161 infants that comprised of birthweight and gender of infants as clinical variables [21].

The nHP population-specific metabolite-only model for post-natal GA assessment in our study revealed an RMSE of 1.33 weeks which is similar to the reported North American population models [21,22]. As our data set included paired nHP and uCB from the same participant, it helped us to compare the RMSEs using metabolites acquired on both types of samples. Our uCB metabolite-only and uCB full models predicted better RMSE when compared to a recent study conducted on an African population using only uCB data set models [44]. Nonetheless, the GARBH-Ini cohort’s nHP full model faired much better and classified up to 92.5% of neonates within ±2 weeks by estimating GA accurately to 1.14 weeks as seen in the earlier published models [21,22,24], while the uCB data set classified only 89.9% of neonates within ±2 weeks by estimating GA accurately to 1.26 weeks.

The estimated GA in our study participants, based on the new Ballards score, revealed an RMSE of ±2 weeks (data not shown) compared to our best fit predicted nHP full model with an RMSE of ±1 week, suggesting a 50% higher error rate in GA estimation using conventional methods, thus, emphasising ML-based methods to be more powerful and independent of intra-observer variability.

A study conducted in LMIC setting reveals that 43.3 and 6.8% of pregnant mothers present themselves at the antenatal care in their second and third trimesters respectively [44]. This is a considerably high number of unbooked pregnant women who directly visit the hospital near to the time of delivery. There is sufficient evidence to suggest that the third-trimester USG with an RMSE of 21–30 days, is the least reliable method of GA assessment near to the time of delivery [44,45], Compared to already published reports, our nHP full model with an RMSE of 1.14 weeks is a better fit for the USG-based GA assessment that is done in these trimesters or near to delivery. Based on our sub-group analysis, we found that the nHP full model performed best in estimating GA on test data sets stratified by different birth outcomes, especially for the TB and non-SGA sub groups compared to the PTB, SGA and LBW. The reason could be due to lesser number of samples from the adverse outcome groups as compared to the healthy outcome groups in our study population. The recent executive summary on SVN, projects that, globally every fourth baby is born too soon and/or born too small. If not addressed in time, these vulnerable infants may be affected in their later life [5,6]. To prevent this global health and economic burden, the need to accurately estimate GA for proper identification and clinical management of these neonates is important. Fortunately, our nHP full model is able to accurately predict GA at delivery in the PTB, SGA, and LBW, with the least error rate of ~ 7–13 days.

To the best of our knowledge, this is the first population-specific post-natal GA assessment model based on metabolic profiles acquired on NBS platforms in an Indian setting. Our models, which estimate GA as close to the actual GA assessed by dating USG provide a proof of concept that may have the potential to be implemented as a metabolic profile-based GA assessment in clinical settings in LMICs.

The significant strength of our study is the ability to compare the estimated GA with the gold standard GA captured by dating USG before 20 weeks in the GARBH-Ini cohort. Accurate measurements of the period of gestation using dating USG are relatively rare in LMIC settings due to the high cost and inaccessibility of the equipment. In this pregnancy cohort, the GA for all new-borns at delivery is also calculated using the new Ballards score. This allowed us to compare the ML-based GA estimations with the conventional methods currently being followed. In our study, we were able to utilise the paired nHP and uCB stored samples from the same newborn. Interestingly, out of the selected features for model-building activities, 16 metabolites in the nHP and uCB data sets were found to be common. These models, may thus, be generalisable for these sample types in the study. Besides, when compared to the best of the ten variables reported from western populations, five of the metabolites were found to be common with our data sets [21]. To date, none of the studies have utilised the placental weight as one of the GA predictors in their population-specific models. Fortunately, we have identified the placental weight as the second-highest predictor in our data set.

The potential limitation of our study is the smaller sample size as compared to the usual sample sizes applied in the ML universe and previously used in Western models for estimating GA at delivery. We could not collect nHP samples from extremely PTB infants as parents refused consent and this reflects the very small numbers of such infants in our model-building strategies. Our maximum study population in the data set is from TB infants which may possibly overestimate the GA measurement in some of the subsets of the models built.

In future, adding extremely PTB infants to the data set may help improve the models to accurately measure GA for the small vulnerable populations. Potential factors that may affect the metabolic profiles, i.e. multiple births, maternal nutrition or medications, environmental exposures during pregnancy, and timing of sample collection need to be considered during model development. We tried to reduce these biases in our model data sets by restricted selection of the study participants.

## CONCLUSIONS

Accurate estimation of post-natal GA is an important guiding factor for physicians to provide best practice in neonatal critical care units at delivery. The XGBoost algorithm applied to the metabolic and clinical parameters of the nHP and uCB data sets offer an accurate GA at delivery in LMICs settings. This postnatal GA model proved far better to the conventional Ballard score based GA method that reduced the error rate by 50% in estimating the predicted GA. As, both the nHP and uCB full models aided in similar identification of PTB at delivery, uCB may be a preferential choice for ease of sampling both for the parents and caregivers. Further, validation of these models in a larger sample size and external cohorts will aid the robustness to estimate the accurate GA and in classification of the birth outcomes especially for unbooked pregnancies. With newborn metabolic testing picking up pace in India, in both government tertiary hospitals and private sectors, the application of these models in clinical settings may improve the GA assessment and aid neonatologists with the effective use of potentially life-saving interventions especially for PTB outcomes. Besides, identification of a handful of the best predictive metabolites could help translatability to clinical settings using point-of-care testing in low-resource settings. By addressing these practical and logistical considerations, ML models can be implemented more effectively in LMIC settings, contributing to improved health care outcomes, resource utilisation and reducing the economic load both for the family and nation. This application may prove useful as a surveillance tool to determine the region-specific PTB rates in LMICs.

## Additional material


Online Supplementary Document

## References

[1] Ohuma EO Bradley E National, regional, and worldwide estimates of preterm birth in 2020, with trends from 2010: a systematic analysis. Lancet. 2023;402:1261–71. 10.1016/S0140-6736(23)00878-4 37805217

[2] WHO. WHO recommendations for care of the preterm or low-birth-weight infant. Geneva; 2022. 36449655

[3] Blencowe H Cousens S Chou D Oestergaard M Say L Moller AB Born too soon: the global epidemiology of 15 million preterm births. Reprod Health. 2013;10 Suppl 1:S2. 10.1186/1742-4755-10-S1-S2 24625129 PMC3828585

[4] Perin J Mulick A Yeung D Villavicencio F Lopez G Strong KL Global, regional, and national causes of under-5 mortality in 2000-19: an updated systematic analysis with implications for the Sustainable Development Goals. Lancet Child Adolesc Health. 2022;6:106–15. 10.1016/S2352-4642(21)00311-4 34800370 PMC8786667

[5] Ashorn P Ashorn U Muthiani Y Aboubaker S Askari S Bahl R Small vulnerable newborns-big potential for impact. Lancet. 2023;401:1692–706. 10.1016/S0140-6736(23)00354-9 37167991

[6] Lawn JE Ohuma EO Bradley E Idueta LS Hazel E Okwaraji YB Small babies, big risks: global estimates of prevalence and mortality for vulnerable newborns to accelerate change and improve counting. Lancet. 2023;401:1707–19. 10.1016/S0140-6736(23)00522-6 37167989

[7] Pusdekar YV Patel AB Kurhe KG Bhargav SR Thorsten V Garces A Rates and risk factors for preterm birth and low birthweight in the global network sites in six low- and low middle-income countries. Reprod Health. 2020;17:187. 10.1186/s12978-020-01029-z 33334356 PMC7745351

[8] Liu L Johnson HL Cousens S Perin J Scott S Lawn JE Global, regional, and national causes of child mortality: an updated systematic analysis for 2010 with time trends since 2000. Lancet. 2012;379:2151–61. 10.1016/S0140-6736(12)60560-1 22579125

[9] Liu L Oza S Hogan D Chu Y Perin J Zhu J Global, regional, and national causes of under-5 mortality in 2000–15: an updated systematic analysis with implications for the Sustainable Development Goals. Lancet. 2016;388:3027–35. 10.1016/S0140-6736(16)31593-8 27839855 PMC5161777

[10] Bhatnagar S Majumder PP Salunke DM A Pregnancy Cohort to Study Multidimensional Correlates of Preterm Birth in India: Study Design, Implementation, and Baseline Characteristics of the Participants. Am J Epidemiol. 2019;188:621–31. 10.1093/aje/kwy284 30770926

[11] Puchalski Ritchie LM Khan S Moore JE Timmings C van Lettow M Vogel JP Low- and middle-income countries face many common barriers to implementation of maternal health evidence products. J Clin Epidemiol. 2016;76:229–37. 10.1016/j.jclinepi.2016.02.017 26931284

[12] Hyder A Lee HJ Ebisu K Koutrakis P Belanger K Bell ML PM2.5 exposure and birth outcomes: use of satellite- and monitor-based data. Epidemiology. 2014;25:58–67. 10.1097/EDE.0000000000000027 24240652 PMC4009503

[13] Arroyo V Díaz J Salvador P Linares C Impact of air pollution on low birth weight in Spain: An approach to a National Level Study. Environ Res. 2019;171:69–79. 10.1016/j.envres.2019.01.030 30660920

[14] Majola L Budhram S Govender V Naidoo M Godlwana Z Lombard C Reliability of last menstrual period recall, an early ultrasound and a Smartphone App in predicting date of delivery and classification of preterm and post-term births. BMC Pregnancy Childbirth. 2021;21:493. 10.1186/s12884-021-03980-6 34233644 PMC8265063

[15] Spinnato JA Sibai BM Shaver DC Anderson GD Inaccuracy of Dubowitz gestational age in low birth weight infants. Obstet Gynecol. 1984;63:491–5. 6700894

[16] Taylor RA Denison FC Beyai S Owens S The external Ballard examination does not accurately assess the gestational age of infants born at home in a rural community of The Gambia. Ann Trop Paediatr. 2010;30:197–204. 10.1179/146532810X12786388978526 20828452 PMC3026295

[17] Ballard JL Khoury JC Wedig K Wang L Eilers-Walsman BL Lipp R New Ballard Score, expanded to include extremely premature infants. J Pediatr. 1991;119:417–23. 10.1016/S0022-3476(05)82056-6 1880657

[18] King JC Physiology of pregnancy and nutrient metabolism. Am J Clin Nutr. 2000;71(5 Suppl):1218S–25S. 10.1093/ajcn/71.5.1218s 10799394

[19] Liang L Rasmussen MH Piening B Shen X Chen S Röst H Metabolic Dynamics and Prediction of Gestational Age and Time to Delivery in Pregnant Women. Cell. 2020;181:1680–92.e15. 10.1016/j.cell.2020.05.002 32589958 PMC7327522

[20] Manuck TA Lai Y Ru H Glover AV Rager JE Fry RC Metabolites from midtrimester plasma of pregnant patients at high risk for preterm birth. Am J Obstet Gynecol MFM. 2021;3:100393. 10.1016/j.ajogmf.2021.100393 33991707 PMC8555706

[21] Ryckman KK Berberich SL Dagle JM Predicting gestational age using neonatal metabolic markers. Am J Obstet Gynecol. 2016;214:515.e1–.e13. 10.1016/j.ajog.2015.11.028 26645954 PMC4808601

[22] Wilson K Hawken S Potter BK Chakraborty P Walker M Ducharme R Accurate prediction of gestational age using newborn screening analyte data. Am J Obstet Gynecol. 2016;214:513.e1–.e9. 10.1016/j.ajog.2015.10.017 26519781

[23] Murphy MS Hawken S Cheng W Wilson LA Lamoureux M Henderson M External validation of postnatal gestational age estimation using newborn metabolic profiles in Matlab, Bangladesh. eLife. 2019;8:e42627. 10.7554/eLife.42627 30887951 PMC6424558

[24] Sazawal S Ryckman KK Mittal H Khanam R Nisar I Jasper E Using AMANHI-ACT cohorts for external validation of Iowa new-born metabolic profiles based models for postnatal gestational age estimation. J Glob Health. 2021;11:04044. 10.7189/jogh.11.04044 34326994 PMC8285766

[25] Hawken S Ward V Bota AB Lamoureux M Ducharme R Wilson LA Real world external validation of metabolic gestational age assessment in Kenya. PLOS Glob Public Health. 2022;2:e0000652. 10.1371/journal.pgph.0000652 36962760 PMC10021775

[26] Lee AC Blencowe H Lawn JE Small babies, big numbers: global estimates of preterm birth. Lancet Glob Health. 2019;7:e2–3. 10.1016/S2214-109X(18)30484-4 30389450

[27] Beretta L Santaniello A Nearest neighbor imputation algorithms: a critical evaluation. BMC Med Inform Decis Mak. 2016;16 Suppl 3:74. 10.1186/s12911-016-0318-z 27454392 PMC4959387

[28] Villar J Cheikh Ismail L Victora CG Ohuma EO Bertino E Altman DG International standards for newborn weight, length, and head circumference by gestational age and sex: the Newborn Cross-Sectional Study of the INTERGROWTH-21st Project. Lancet. 2014;384:857–68. 10.1016/S0140-6736(14)60932-6 25209487

[29] Kursa M Rudnicki W Feature Selection with Boruta Package. J Stat Softw. 2010;36:1–13. 10.18637/jss.v036.i11

[30] Arlot S Celisse A A Survey of Cross Validation Procedures for Model Selection. Stat Surv. 2009;4:40–79.

[31] Abhishek B, Tyagi AK, editors. An Useful Survey on Supervised Machine Learning Algorithms: Comparisons and Classifications. Advances in Electrical and Computer Technologies. Singapore: Springer Nature Singapore; 2022.

[32] Landset S Khoshgoftaar TM Richter AN Hasanin T A survey of open source tools for machine learning with big data in the Hadoop ecosystem. J Big Data. 2015;2:24. 10.1186/s40537-015-0032-1

[33] Hoffman M Liu W Tunguhan J Bitar G Kumar K Ewen E Machine Learning Algorithm Using Clinical Data and Demographic Data for Preterm Birth Prediction. Am J Obstet Gynecol. 2022;226:S362–3. 10.1016/j.ajog.2021.11.608

[34] Wen JY Liu CF Chung MT Tsai YC Artificial intelligence model to predict pregnancy and multiple pregnancy risk following in vitro fertilization-embryo transfer (IVF-ET). Taiwan J Obstet Gynecol. 2022;61:837–46. 10.1016/j.tjog.2021.11.038 36088053

[35] Bai X Zhou Z Luo Y Yang H Zhu H Chen S Development and Evaluation of a Machine Learning Prediction Model for Small-for-Gestational-Age Births in Women Exposed to Radiation before Pregnancy. J Pers Med. 2022;12:550. 10.3390/jpm12040550 35455666 PMC9031835

[36] Chen T, Guestrin C. XGBoost: A Scalable Tree Boosting System. Proceedings of the 22nd ACM SIGKDD International Conference on Knowledge Discovery and Data Mining; San Francisco, California, USA: Association for Computing Machinery; 2016. p. 785–94.

[37] Robin X Turck N Hainard A Tiberti N Lisacek F Sanchez JC pROC: an open-source package for R and S+ to analyze and compare ROC curves. BMC Bioinformatics. 2011;12:77. 10.1186/1471-2105-12-77 21414208 PMC3068975

[38] DeLong ER DeLong DM Clarke-Pearson DL Comparing the areas under two or more correlated receiver operating characteristic curves: a nonparametric approach. Biometrics. 1988;44:837–45. 10.2307/2531595 3203132

[39] Zhang Y Sylvester KG Jin B Wong RJ Schilling J Chou CJ Development of a Urine Metabolomics Biomarker-Based Prediction Model for Preeclampsia during Early Pregnancy. Metabolites. 2023;13:715. 10.3390/metabo13060715 37367874 PMC10301596

[40] Wang X Wang Y Zhang S Yao L Xu S Analysis and Prediction of Gestational Diabetes Mellitus by the Ensemble Learning Method. International Journal of Computational Intelligence Systems. 2022;15:72. 10.1007/s44196-022-00110-8

[41] Han JH Yoon SJ Lee HS Park G Lim J Shin JE Application of Machine Learning Approaches to Predict Postnatal Growth Failure in Very Low Birth Weight Infants. Yonsei Med J. 2022;63:640–7. 10.3349/ymj.2022.63.7.640 35748075 PMC9226835

[42] Easton ZJW Regnault TRH The Impact of Maternal Body Composition and Dietary Fat Consumption upon Placental Lipid Processing and Offspring Metabolic Health. Nutrients. 2020;12:3031. 10.3390/nu12103031 33022934 PMC7601624

[43] Jasper EA Oltman SP Rogers EE Dagle JM Murray JC Kamya M Targeted newborn metabolomics: prediction of gestational age from cord blood. Journal of perinatology: official journal of the California Perinatal Association. 2022;42:181–6. 10.1038/s41372-021-01253-w 35067676 PMC8830770

[44] Jiwani SS Amouzou-Aguirre A Carvajal L Chou D Keita Y Moran AC Timing and number of antenatal care contacts in low and middle-income countries: Analysis in the Countdown to 2030 priority countries. J Glob Health. 2020;10:010502. 10.7189/jogh.10.010502 32257157 PMC7101027

[45] Smith GCS Nakimuli A Ultrasound estimation of gestational age in late pregnancy in low-income countries: made to measure or off-the-peg? Lancet Glob Health. 2020;8:e462–3. 10.1016/S2214-109X(20)30081-4 32199109

