# Supplementary material for: Post-natal gestational age assessment using targeted metabolites of neonatal heel prick and umbilical cord blood: A GARBH-Ini cohort study from North India
Source: J Glob Health. 2024 Jul 5;14:04115. doi: 10.7189/jogh.14.04115 (PMC11225965; doi:10.7189/jogh.14.04115)
Supplement: Online Supplementary Document [file jogh-14-04115-s001.pdf]

Supplementary Figure 1. Overview of study design and population-specific model development for post-natal GA prediction

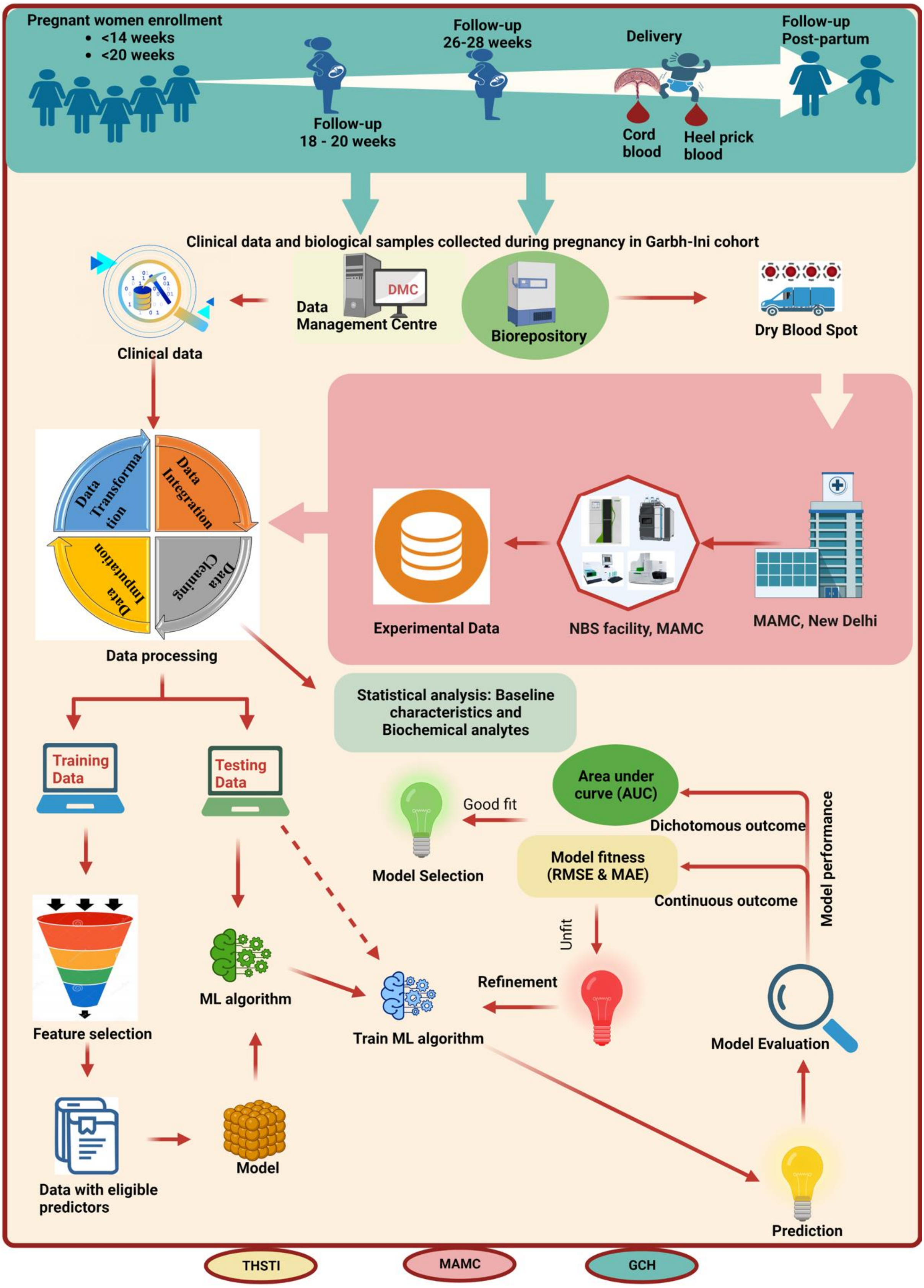

Supplementary Table 1. List of the independent variables used for feature selection and list of selected predictors used for nHP and uCB model building datasets.

Class of variables	List of variables of HP and CB used for FS (n=47)	Linear and Squared term in HP model (31)	Linear and Squared term in CB model (24)
Amino acids (11)	Alanine, Arginine, Leucine, Methionine, Phenylalanine, Tyrosine, Valine, Proline, Citrulline, Glycine, and Ornithine (11)	Alanine, Glycine, Leucine, Tyrosine, Proline, Valine, Ornithine, and Citrulline (8)	Alanine, Arginine, Glycine, Leucine, Tyrosine and, Valine (6)
Acylcarnitines (30)	Free carnitine (C0), Acetyl carnitine (C2), Propionyl carnitine (C3), Malonyl carnitine (C3-DC), Butyryl carnitine (C4), Isovalerylcarnitine (C5), Tiglylcarnitine (C5:1), succinylcarnitine (C4-DC), 3-HO-isovaleryl carnitine (C5DC), Hexanoyl carnitine (C6), Methylglutaryl carnitine (C6-DC), Octanoyl carnitine (C8), Octenoyl carnitine (C8:1), Decanoyl carnitine (C10), Decenoyl carnitine (C10:1), Decadienyl carnitine (C10:2), Dodecanoyl carnitine (C12), Dodecenoyl carnitine (C12:1), Tetradecanoyl carnitine (C14), Tetradecenoyl carnitine (C14:1), 3-HO-tetradecanoyl carnitine (C14-OH), Palmitoyl carnitine (C16),	Acetyl carnitine (C2), Propionyl carnitine (C3), Malonyl carnitine (C3-DC), succinylcarnitine (C4-DC), Isovaleryl carnitine (C5), 3-HO-isovaleryl carnitine (C5DC), Hexanoyl carnitine (C6), Dodecanoyl carnitine (C12), Dodecenoyl carnitine (C12:1), Tetradecanoyl carnitine (C14), Tetradecenoyl carnitin (C14:1), Palmitoyl carnitine (C16), Palmitoleyl carnitine (C16:1), 3-HO-palmitoyl carnitine (C16-OH), 3-OH-palmitoleyl carnitine (C16:1-OH), Stearoyl carnitine (C18), Oleoyl carnitine	Acetyl carnitine (C2), Propionyl carnitine (C3), succinylcarnitine (C4-DC), Isovaleryl carnitine (C5), Hexanoyl carnitine (C6), Decanoyl carnitine (C10), Dodecanoyl carnitine (C12), Tetradecenoyl carnitin (C14:1), Palmitoyl carnitine (C16), Palmitoleyl carnitine (C16:1), 3-OH-palmitoleyl carnitine (C16:1-OH), Stearoyl carnitine (C18), Oleoyl carnitine (C18:1), 3-Hydroxyoleoyl carnitine (C18:1OH), Linoleoylcarnitine (C18:2) (15)

Class of variables	List of variables of HP and CB used for FS (n=47)	Linear and Squared term in HP model (31)	Linear and Squared term in CB model (24)
	Palmitoleyl carnitine (C16:1), 3-HO-palmitoyl carnitine (C16-OH), 3-Hydroxypalmitoleyl carnitine (C16:1-OH), Stearoyl carnitine (C18), Oleoyl carnitine (C18:1), OH-stearoyl carnitine (C18OH), 3-Hydroxyoleoyl carnitine (C18:1OH), Linoleoylcarnitine (C18:2) (30)	(C18:1), OH-stearoyl carnitine (C18OH), 3-Hydroxyoleoyl carnitine (C18:1OH), Linoleoylcarnitine (C18:2) (20)	
Clinical factors (3)	Placental weight, gender of baby, birth weight (3)	Placental weight and Birth weight (2)	Placental weight and Birth weight (2)
Enzyme and Hormones (3)	Galactose-1 Phosphate Uridyl Transferase (GALT), 17-Hydroxyprogesterone (17-OHP), Thyroid Stimulating Hormone (TSH) (3)	17-OHP (1)	GALT (1)

Supplementary Figure 2 A & B. Selection of predictors using Boruta algorithm from the training dataset of neonatal HP and umbilical CB

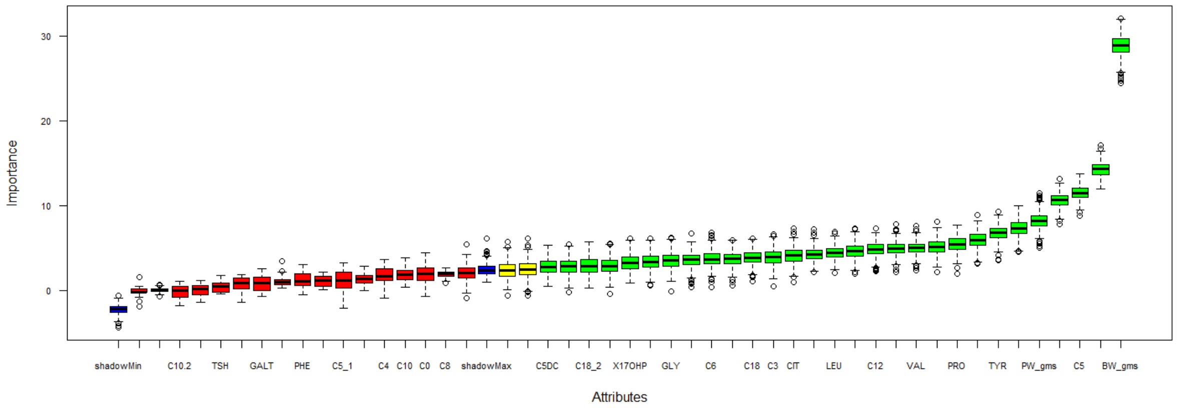

A) nHP dataset: Different colours of box plot depicts the output of feature selection that shows the degree of relative variable importance with 95% CI. In this plot, 29 confirmed (green box), 2 tentative (yellow box), and 16 unconfirmed (red box) features were classified. Blue box plots are shadow attributes that represent the minimum, average, and maximum value that was used to select the features.

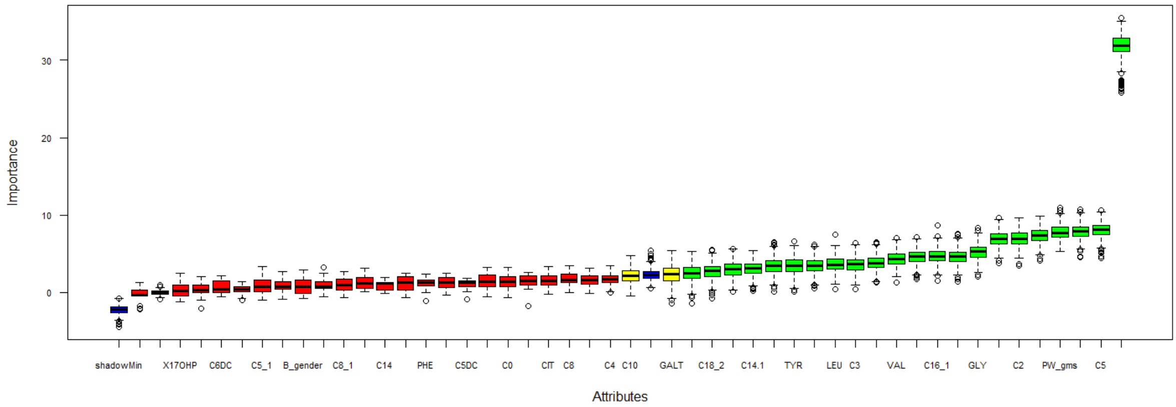

B) uCB dataset: In this plot, 22 confirmed (green box), 2 tentative (yellow box), and 23 unconfirmed (red box) features were classified.

Supplementary Table 2 A. Univariate analysis comparing the mean differences of the selected predictors between PTB and TB in nHP dataset*

Analytes	PTB, Mean±SD	95% CI	TB, Mean±SD	95% CI	p-value
**Birth weight**	-1.2117±0.8	-1.3738-(-1.0496)	0.0995±0.9	0.4537-0.1536	**0.000**
**Placental weight**	-0.4142 ± 0.9	-0.5946 – (-0.2338)	0.034 ± 0.98	-0.0231 - 0.0911	**0.000**
**17-OHP**	-0.0409±0.69	-0.1805-0.0986	0.0033±1.02	-0.0549-0.0616	0.56
**Alanine**	-0.2335 ± 1.13	-0.4639 – (-0.0052)	0.0192 ± 0.98	-0.037 - 0.0755	**0.035**
**Citrulline**	-0.0292 ± 1.53	-0.3389 - 0.2805	0.0023 ± 0.94	-0.0514 - 0.0562	0.84
**Ornithine**	-0.1485±1.02	-0.3542-0.0576	0.0121±0.03	-0.0448-0.0691	0.13
**Leucine**	0.1134± 0.98	0.0848 - 0.3118	0.0093 ±1.00	-0.0664 - 0.0478	0.24
**Glycine**	-0.1650±0.88	-0.3436-0.0135	0.0135±1.01	-0.0439-0.0710	0.09
**Valine**	0.0055 ± 0.97	-0.2030 - 0.1919	0.0004 ± 1.00	-0.0567 - 0.0576	0.95
**Tyrosine**	0.4655 ± 1.49	0.1638 - 0.7672	-0.0382 ±0.93	-0.0918 - 0.0153	**0.001**
**Proline**	0.0184 ± 1.11	-0.206 - 0.2429	-0.0015±0.99	-0.058 - 0.0550	0.86
**C2**	-0.2037 ± 0.92	-0.3900 - 0.0175	0.0167±1.00	-0.0406 - 0.0740	**0.02**
**C3**	0.1023 ± 1.18	-0.1366 - 0.3412	0.0084±0.98	-0.0645 - 0.0477	0.29
**C3DC**	0.1793± 2.13	-0.2509 - 0.6097	0.0147±0.84	-0.0627 - 0.0333	0.37
**C4DC**	-0.2455 ± 0.99	-0.4452 – (-0.0458)	0.0201±0.99	-0.0368 - 0.0771	**0.012**
**C5**	0.6579 ± 1.43	0.3692 - 0.9466	-0.0540 ±0.93	-0.1074 - 0.0005	**0.0001**
**C5DC**	0.0947±1.42	-0.1914-0.381	-0.0077±0.95	-0.0624-0.0468	0.33
**C6**	0.0299±0.98	-0.2283-0.1684	-0.0024±1.0	- 0.0573-0.0596	0.76
**C12**	0.1705 ± 1.11	-0.0547 - 0.3959	-0.0140 ±0.98	-0.0704 - 0.4244	0.12
**C12:1**	0.1130 ± 1.13	-0.1160 - 0.3421	-0.0092±0.98	-0.0656 - 0.0471	0.31
**C14**	0.2504 ± 1.22	-0.0001 - 0.5010	-0.0205±0.97	-0.0762 - 0.0351	**0.039**
**C14:1**	0.2415 ± 1.27	0.0151 - 0.4981	0.0198±0.97	0.0753 - 0.0356	**0.05**
**C16**	0.1506 ± 0.48	0.0526 - 0.2486	-0.0123±1.03	-0.0711 - 0.0464	**0.005**
**C16: OH**	0.1566±1.06	-0.0581-0.3713	- 0.0128±0.99	-0.0696-0.0439	0.11
**C16:1**	0.0104 ± 1.04	-0.1994 - 0.2202	-0.00080±0.99	-0.0577 - 0.0560	0.9182
**C16:1OH**	-0.0556± 0.63	-0.1841-0.0728	0.0045± 1.02	-0.0538-0.0630	0.57
**C18**	0.0246 ± 0.98	-0.1730 - 0.2223	-0.0020±1.00	-0.0592 - 0.0551	0.8
**C18:1**	-0.0747 ± 0.65	-0.2057 - 0.0562	0.0061±1.02	-0.0522 - 0.0645	0.26
**C18OH**	0.0165 ± 0.72	-0.1287 - 0.1617	-0.0013±1.01	-0.0595 - 0.0568	0.82
**C18:1OH**	0.0119 ± 1.2	-0.2310 - 0.2549	-0.0009±0.98	-0.0503 - 0.0551	0.9
**C18:2**	-0.0077±0.8	-0.1687-0.1532	0.0006±1.01	-0.0573-0.0585	0.13

SD=Standard Deviation, PTB=Preterm birth, TB=Term birth, 95%CI=95% confidence interval; *All variables are z-score transformed

Supplementary Table 2 B. Univariate analysis comparing the mean differences of the selected predictors between PTB and TB in uCB dataset*

Analytes	PTB, Mean±SD	95% CI	TB, Mean±SD	95% CI	p-value
**Birth weight**	-1.2117±0.80	-1.3738-(-1.0496)	0.0995±0.90	0.4537-0.1536	**0.000**
**Placental weight**	-0.4142 ± 0.90	-0.5946 – (-0.2338)	0.034 ± 0.98	-0.0231 - 0.0911	**0.000**
**GALT**	-0.2054±1.05	-0.4174-0.0065	0.0169±0.99	-0.0398-0.0736	**0.03**
**Alanine**	-0.2102±1.15	-0.4426-0.0220	0.0172±0.98	-0.0389-0.0735	**0.03**
**Leucine**	-0.1508±1.06	-0.3640-0.0623	0.0129±0.99	-0.0443-0.0691	0.11
**Glycine**	0.1650±0.88	-0.3436-0.0135	0.0135±0.93	-0.0439-0.0711	0.09
**Arginine**	0.1912±0.09	-0.0041-0.3782	0.0137±0.90	-0.0731-0.0416	**0.05**
**Tyrosine**	0.1518±1.15	-0.0807-0.3843	-0.0124±0.98	-0.0687-0.0438	0.12
**Valine**	-0.0580±1.13	-0.286-0.1701	0.0048±0.98	-0.0516-0.0612	0.5
**C2**	-0.1931±0.84	-0.3636-(-0.0226)	0.0158±1.01	-0.0418-0.0735	**0.04**
**C3**	-0.0828±0.90	-0.2656-0.1001	0.0068±1.01	-0.0507-0.0643	0.39
**C4DC**	-0.2943±0.84	-0.4643-(-0.1243)	0.0241±1.01	-0.0333-0.0817	**0.002**
**C5**	0.2836±0.84	0.0762-0.4910	- 0.0232±1.01	-0.0800-0.0334	**0.003**
**C6**	0.0965±0.90	-0.0841-0.2771	- 0.0079±1.01	-0.0655-0.4962	0.32
**C10**	0.0697±0.90	-0.1114-0.2507	- 0.0057±1.08	- 0.0632-0.5182	0.47
**C12**	0.1336±1.18	-0.1043-0.3717	- 0.0109±0.98	- 0.0671-0.0451	0.17
**C14:1**	0.1951±1.28	-0.0634-0.4538	-0.0160±0.97	-0.0715-0.0394	**0.04**
**C16**	0.1552±1.03	-0.0525-0.3630	- 0.0127±0.99	-0.0696-0.0441	0.11
**C16:1**	0.0380±1.12	-0.1883-0.2643	- 0.0031±0.98	-0.0596-0.0533	0.69
**C16:1OH**	-0.3144±0.78	-0.4718-(-0.1570)	0.0258±1.01	-0.0319-0.0835	**0.001**
**C18**	-0.0449±0.87	-0.2223-0.1323	0.0036±1.01	-0.0539-0.0613	0.64
**C18:2**	0.1204±1.89	-0.2601-0.5007	-0.0098±0.89	- 0.0607-0.0408	0.28
**C18:1**	0.0997±1.56	-0.2144-0.4138	- 0.0081±0.95	-0.0618-0.0454	0.31
**C18:1OH**	-0.2952±0.92	-0.4810-(-0.1095)	0.0242±1.01	-0.0329-0.0814	**0.002**

SD=Standard Deviation, PTB=Preterm birth, TB=Term birth, 95%CI=95% confidence interval; *All variables are z-score transformed.

Supplementary Table 3. Development of six different models using the selected predictors.

Model	No. of variables included and their sources in the models
Birth weight (M1)	Only birthweight (1 linear and 1 squared term), a total of 2 variables were considered.
Baseline clinical (M2)	Birth weight and placental weight (2 linear and 2 squared terms), a total of 4 variables were considered.
Heel prick (M3)	Neonatal heel prick analytes only, (29 linear and 29 squared terms), a total of 58 variables were considered.
Cord blood (M4)	Umbilical cord blood analytes only, (22 linear and 22 squared terms), a total of 44 variables were considered.
HP full (M5)	Birthweight, placental weight, and neonatal heel prick analytes, (31 linear and 31 squared terms), a total of 62 variables were considered.
CB full (M6)	Birthweight, placental weight, and umbilical cord blood analytes, (24 linear and 24 squared terms), a total of 48 variables were considered.

Supplementary Table 4: Overall model performance for GA estimation and birth outcome as PTB and TB discrimination on test datasets in days.

Model	No. of predictors (linear, squared)	RMSE (in days)	MAE (in days)	AUC (95% CI)
**Baby weight (M1)**	2 (1,1)	9.1	7.07	0.85 (0.81-0.91)
**Baseline clinical (M2)**	4 (2,2)	8.96	7.07	0.87 (0.82-0.92)
**Neonatal HP (M3)**	58 (29,29)	9.31	7.49	0.73 (0.64-0.82)
**Umbilical CB (M4)**	44 (22,22)	10.22	7.91	0.69 (0.59-0.78)
**HP full model (M5)**	62 (31,31)	7.98	6.37	0.89 (0.84-0.94)
**CB full model (M6)**	48 (24,24)	8.82	6.86	0.89 (0.85-0.95)

RMSE= root mean squared error; MAE=mean absolute error; AUC=area under ROC curve

Supplementary Table 5. The frequency of concordance between dating ultrasound-based GA and ML-based GA in nHP full (M5) and uCB full (M6) models

	n	≤ 34 wks	35-36 wks	37-38 wks	39-40 wks	>40 wks
**Heel Prick**						
**≤ 34 wks**	4	0 (0%)	3	1	-	-
**35-36 wks**	27	1	3(11.11%)	22	1	-
**37-38 wks**	108	-	3	60(55.55%)	45	-
**39-40 wks**	197	-	-	67	129 (65.48%)	1
**>40 wks**	38	-	-	4	34	0 (0%)
**Cord Blood**						
**≤ 34 wks**	4	0 (0%)	3	1	-	-
**35-36 wks**	27	-	1 (3.7%)	24	2	-
**37-38 wks**	108	-	5	64 (59.26%)	42	-
**39-40 wks**	197	-	-	49	147(74.62%)	1
**>40 wks**	38	-	-	10	28	0 (0%)
